# WGCNA-ML-MR integration: uncovering immune-related genes in prostate cancer

**DOI:** 10.3389/fonc.2025.1534612

**Published:** 2025-04-07

**Authors:** Jing Lv, Yuhua Zhou, Shengkai Jin, Chaowei Fu, Yang Shen, Bo Liu, Menglu Li, Yuwei Zhang, Ninghan Feng

**Affiliations:** ^1^ Wuxi School of Medicine, Jiangnan University, Wuxi, China; ^2^ Department of Urology, Jiangnan University Medical Center, Wuxi, China; ^3^ Department of Clinical Medicine, Nanjing Medical University, Nanjing, China; ^4^ Department of Clinical Medicine, Nantong University Medical School, Nantong, China

**Keywords:** prostate cancer, machine learning, weighted gene co-expression network analysis, Mendelian randomization, biomarkers

## Abstract

**Background:**

Prostate cancer is one of the most common tumors in men, with its incidence and mortality rates continuing to rise year by year. Prostate-specific antigen (PSA) is the most commonly used screening indicator, but its lack of specificity leads to overdiagnosis and overtreatment. Therefore, identifying new biomarkers related to prostate cancer is crucial for the early diagnosis and treatment of prostate cancer.

**Methods:**

This study utilized datasets from the Gene Expression Omnibus (GEO) to screen for differentially expressed genes (DEGs) and employed Weighted Gene Co-expression Network Analysis (WGCNA) to identify driver genes highly associated with prostate cancer within the modules. The intersection of differentially expressed genes and driver genes was taken, and Kyoto Encyclopedia of Genes and Genomes (KEGG) and Gene Ontology (GO) enrichment analyses were performed. Furthermore, a machine learning algorithm was used to screen for core genes and construct a diagnostic model, which was then validated in an external validation dataset. The correlation between core genes and immune cell infiltration was analyzed, and Mendelian randomization (MR) analysis was conducted to identify biomarkers closely related to prostate cancer.

**Results:**

This study identified six core biomarkers: SLC14A1, ARHGEF38, NEFH, MSMB, KRT23, and KRT15. MR analysis demonstrated that MSMB may be an important protective factor for prostate cancer. In q-PCR experiments conducted on tumor tissues and adjacent non-cancerous tissues from prostate cancer patients, it was found that: compared to the adjacent non-cancerous tissues, the expression level of ARHGEF38 in prostate cancer tumor tissues significantly increased, while the expression levels of SLC14A1, NEFH, MSMB, KRT23, and KRT15 significantly decreased. To further validate these findings at the protein level, we conducted Western blot analysis, which corroborated the q-PCR results, demonstrating consistent expression patterns for all six biomarkers. IHC results confirmed that ARHGEF38 protein was highly expressed in tumor tissues, while MSMB expression was markedly reduced.

**Conclusion:**

Our study reveals that SLC14A1, ARHGEF38, NEFH, MSMB, KRT23, and KRT15 are potential diagnostic biomarkers for prostate cancer, among which MSMB may play a protective role in prostate cancer.

## 1 Introduction

Prostate cancer (PCa) is the second most common cancer globally and the fifth leading cause of cancer death among men (1). Statistics show that the incidence of prostate cancer is gradually increasing every year, especially in developed countries, partly due to an aging population and changes in lifestyle (2). Early-stage prostate cancer is usually asymptomatic, and when symptoms appear, the disease often has progressed to a point where it is incurable. Over the past decade, the incidence of PCa has risen significantly while mortality has declined, mainly due to the use of prostate-specific antigen (PSA) as a biomarker for PCa screening (3). Therefore, routine screening and monitoring based on reliable biomarkers are crucial for the early detection and assessment of tumor progression in prostate cancer, which can significantly improve patient survival rates and quality of life.

PCa is typically diagnosed through digital rectal examination, PSA screening, and biopsy. Early studies have shown that PSA is more sensitive than palpable tumor assessment by digital rectal examination in the detection of prostate cancer, with a higher detection rate, and plays an important role in monitoring the prognosis and recurrence of tumor patients (4, 5). Data from the European Randomized Study of Prostate Cancer Screening (ERSPC) indicates that after 16 years of follow-up, PSA screening significantly reduces the mortality rate of PCa, and the longer the follow-up, the greater the reduction in PCa mortality (6). Although PSA as a routine screening indicator has been widely accepted, its lack of specificity may lead to excessive examination and treatment, or overlook some potential malignant lesions (7, 8). Biopsy is the “gold standard” for the diagnosis of prostate cancer, but it may bring complications, including infection, hematospermia, hematuria, urinary retention, and erectile dysfunction (9). Therefore, there is an increasing clinical need to identify new biomarkers for prediction, diagnosis, and prognosis. These biomarkers could complement or reduce the reliance on invasive diagnostic procedures like biopsies.

In recent years, advancements in immunological research have revealed the critical role of immune cells in the occurrence and development of PCa. The infiltration of immune cells not only significantly affects the formation and maintenance of the tumor microenvironment but also plays a decisive role in the biological behaviors of tumor growth, invasion, and metastasis (10). For instance, regulatory T cells (Treg cells) play an important role in regulating tumor progression by modulating immune suppression. Flammiger et al. detected Tregs in PCa specimens through FOXP3 immunohistochemistry and found that Tregs within tumor tissues were positively correlated with PCa staging and the Ki67 index (11). Zhao et al.’s research indicated that in the bone marrow microenvironment of patients with bone metastases, the number of Treg significantly increased. In particular, CD4+CD25^high^ Treg cells may promote the transfer of tumor cells to the skeleton by suppressing the immune microenvironment within the bone marrow (12). Furthermore, Yang et al.’s study demonstrated that in patients with high-risk PCa who underwent radical prostatectomy, the infiltration level of CD8+ T cells within the tumor was independently associated with a significant increase in patient survival rates (13). Therefore, immunotherapy is extremely important in the treatment of PCa. However, compared to other malignant tumors, the clinical efficacy of immunotherapy in PCa has certain limitations (14). In summary, although immunotherapy has shown potential in the treatment of PCa, it still faces many challenges. Future research needs to further explore new biomarkers to provide new immune treatment plans for PCa.

In this study, we obtained 5 publicly available PCa datasets from the Gene Expression Omnibus (GEO) database. Two of these datasets were merged to create a meta-data cohort, which was used as the training group. The remaining three datasets were merged into another meta-data cohort and used as the validation group. We first compared 189 PCa cases with 63 normal control cases in the training group to identify differentially expressed genes (DEGs) and performed principal component analysis (PCA) to eliminate batch effects. Weighted Gene Co-expression Network Analysis (WGCNA) was used to select important module genes. DEGs were then intersected with module genes, and functional enrichment analysis and KEGG enrichment analysis were performed. Machine learning techniques were subsequently employed to screen and identify diagnostic biomarkers for PCa, which were validated in the trial group. Next, we assessed the diagnostic efficacy of the selected diagnostic markers using the receiver operating characteristic (ROC) curve. GSVA analysis was used to analyze the pathways that these genes might enrich, and immune cell infiltration analysis was conducted to screen for core immune-related diagnostic biomarkers for PCa. Finally, we used Mendelian randomization studies to clarify the causal relationship between core biomarkers and PCa. This study aims to provide new molecular markers for the early diagnosis and treatment of PCa and to provide a scientific basis for the early diagnosis and personalized treatment of PCa.

## 2 Materials and methods

### 2.1 Data collection and processing

All PCa datasets were obtained from the GEO public database (http://www.ncbi.nlm.nih.gov/geo) including the GSE46602, GSE79021, GSE200879, GSE60329, and GSE71016 datasets. These datasets were selected based on their inclusion of both tumor and normal prostate tissue samples, as well as their relevance to prostate cancer biomarker discovery. These datasets were divided into a training group (GSE46602 and GSE79021) with 189 PCa cases and 63 normal control cases, and the test group (GSE200879, GSE60329, and GSE71016) with 216 PCa cases and 70 normal control cases. Detailed information about each dataset, including sample collection methods, sources, purposes, and database curators, is provided in **Table 1**. Hyperlinks to the original datasets are included for further reference.

**Table 1:** Fundamental details of the GEO datasets utilized in this research.

GEO series	Platform	Country	Samples		Category
			PCa	Normal	
GSE46602	GPL570	Denmark	36	14	Train group
GSE79021	GPL19370	USA	153	49	Train group
GSE200879	GPL32170	France	115	9	Test group
GSE60329	GPL14550	Italy	54	14	Test group
GSE71016	GPL16699	USA	48	47	Test group

### 2.2 Identification of DEGs in PCa

The raw data were processed into standardized data using the R package “limma”. We screened data from 189 PCa cases and 63 normal control cases in the training group and employed the “SVA” package to correct for batch effects. The threshold for DEG selection was set as |logFold Change (logFC)| > 1 and adjusted p-value < 0.05. Volcano plots for all DEGs and clustering heatmaps were generated using the R packages “ggplot2” and “pheatmap”, respectively.

### 2.3 Weighted gene co-expression network analysis

The R package “WGCNA” is used to construct a gene co-expression network and identify modules related to PCa. A subset of genes with a standard deviation greater than 0.5 is selected for further analysis. The “goodSamplesGenes” function is employed to check for missing values in the data, to remove genes or samples that do not meet the quality standards, and to build a scale-free co-expression network. The “pickSoftThreshold” function is utilized to determine the optimal soft threshold. On this basis, the gene expression data matrix is converted into the corresponding adjacency matrix, which is then transformed into a topological overlap matrix to determine the gene weights and similarities. Gene modules are identified through topological overlap clustering methods. The module eigengenes (ME) are calculated and merged among similar modules, followed by the generation of a hierarchical clustering dendrogram. Subsequently, the Gene significance (GS) and Module Membership (MM) are computed within the modules through intramodular analysis.

### 2.4 Functional enrichment analysis

Using the R package “clusterProfiler,” core genes were subjected to Gene Ontology (GO) analysis and Kyoto Encyclopedia of Genes and Genomes (KEGG) enrichment analysis, with a p-value of <0.05 set as the criterion. To further explore the signaling pathways involved in core differential genes, we employed the Gene Set Variation Analysis (GSVA) method. The R packages “GSVA” and “enrichplot” were used for GSVA analysis of the differential genes, referring to the gene set file “c2.cp.kegg.Hs.symbols.gmt”.

### 2.5 Construction of a diagnostic model using machine learning methods

Machine learning is a branch of artificial intelligence, and the core of machine learning is the algorithms, which can analyze and extract patterns from data and then use these patterns to make predictions or decisions without explicit programming (15). We divided the PCa dataset into a training group and a test group and included 12 machine learning methods, including SVM, Ridge, Enet, glmBoost, Lasso, plsRglm, Stepglm, RF, GBM, LDA, XGBoost, and NaiveBayes. We constructed models with 113 combinations of the 12 algorithms in the PCa training group dataset, and all constructed models were evaluated in the PCa test group dataset. For each model, we calculated its AUC values in the training and validation groups and extracted the feature genes screened by each method. Then, we ranked the predictive performance of the models based on the average AUC values to select the optimal model and the optimal model genes.

### 2.6 Analysis of immune cell infiltration

The CIBERSORT algorithm was utilized to assess the composition and abundance of 22 types of immune-infiltrating cells in both PCa and control samples. The R package “Cibersort” was employed for the immune cell infiltration analysis. Bar charts were used to visualize the proportion of each type of immune cell in different samples. Comparisons in the proportions of different types of immune cells between the PCa and control groups were visualized using the R package “ggpubr”. A heatmap depicting the correlations of the 22 infiltrating immune cells was plotted using the R package “corrplot”.

### 2.7 Correlation analysis between gene identification and immune cell infiltration

We conducted Spearman’s rank correlation analysis using R software to examine the correlation between the expression levels of the identified biomarkers and the levels of infiltrating immune cells. Finally, the results of the analysis were visualized using the R packages “ggpubr” and the “linkET” function.

### 2.8 Mendelian randomization analysis

We obtained expression quantitative trait locus (eQTL) data from the IEU Open GWAS project (https://gwas.mrcieu.ac.uk/), which covers 31,684 blood and peripheral blood mononuclear cell samples involving 19,942 genes. Concurrently, we obtained protein quantitative trait locus (pQTL) data from https://www.decode.com/summarydate/, encompassing 4,907 proteins in the plasma of 35,559 Icelandic individuals, and identified 18,084 sequence variations associated with plasma protein levels. We used these two datasets as exposure data for subsequent Mendelian Randomization (MR) analysis. Furthermore, the PCa outcome data “Finngen_R10_C3_PROSTATE” for MR analysis was derived from https://www.finngen.fi/en/, including 15,199 PCa cases and 131,266 control cases. In our study, single nucleotide polymorphisms (SNPs) were defined as instrumental variables (IVs), with the selection criteria for IVs being: (a) demonstrating a genome-wide significant association (P < 5 × 10^–8^) (16); (b) showing an independent association (linkage disquilibrium (LD) > 10,000 kb, clump r2 < 0.001) (17). Additionally, to avoid bias caused by weak instruments, we considered those IVs with an F statistic >10 as strong instruments and reserved them for the following analysis (18). In the causal inference of Mendelian Randomization (MR), valid instrumental variables (IVs) must satisfy three fundamental assumptions: (1) the genetic variant is directly related to the exposure; (2) the genetic variant is unrelated to potential confounders between the exposure and the outcome; (3) the genetic variant does not affect the outcome through pathways other than the exposure.

### 2.9 Ethical statement and tissue collection

This study was approved by the Ethics Committee of Wuxi No. 2 Hospital affiliated with Nanjing Medical University (2022-Y-80). The PCa specimens and their paired normal tissues were obtained from patients at Wuxi No. 2. Hospital and all participants have signed informed consent forms. All patients received no endocrine therapy before surgery, and all patients underwent radical prostatectomy. The tissues were immediately preserved in liquid nitrogen.

### 2.10 Reagents

FastPure Cell/Tissue Total RNA Isolation Kit V2, HiScript III RT SuperMix, and ChamQ Universal SYBR qPCR Master Mix Q711-02 were all acquired from Vazyme (Nanjing, China). RIPA lysis buffer was purchased from Beyotime (Shanghai, China). Protease Inhibitor Cocktails (Cat. HY-K0010) were sourced from MedChemExpress (Shanghai, China). Anti- ARHGEF38 (Cat. ab122345) was purchased from Abcam(Shanghai, China). Antibodies against UT-B (Cat. 25962-1-AP), NEFH (Cat. 18934-1-AP), MSMB (Cat. 15888-1-AP), KRT23 (Cat. 24049-1-AP), KRT15 (Cat. 10137-1-AP) and Beta Actin (Cat. 66009-1-Ig) were purchased from Proteintech (Wuhan, China). Secondary antibodies (Cat. SA00001-1and Cat. SA00001-2) were purchased from Proteintech (Wuhan, China).

### 2.11 Total RNA extraction and quantitative real-time PCR

Total RNA was isolated from cells with FastPure Tissue Total RNA Isolation Kit V2 and reverse-transcribed into complementary DNAs (cDNAs) with HiScript III RT SuperMix. The cDNAs were amplified based on the standard qPCR protocol with ChamQ Universal SYBR qPCR Master Mix in an Applied Biosystems 7500 Fast Real-Time PCR System (Thermofisher, US). Quantitative PCR was conducted at 95 °C for 30 s, followed by 40 cycles of 95 °C for 10 s and 60 °C for 30 s. GAPDH was used as the internal control and the results were calculated using 2^-ΔΔCT^ method. The sequences of primers can be seen in **Supplementary Table 1**.

### 2.12 Western blot assay

RIPA lysis buffer containing protease inhibitor was used to extract tissue protein. Protein concentration was determined with BCA Protein Assay Kit. ColorMixed Protein Marker was applied as a protein size marker. Total proteins were separated by 10% sodium dodecyl sulfate–polyacrylamide gel electrophoresis and then transferred to a polyvinylidene difluoride (PVDF) membrane (3010040001, Roche, Germany). The PVDF membrane was then blocked with 5% milk for 2 h followed by incubation with the indicated primary antibody overnight at 4°C. After incubation with the corresponding secondary antibody at room temperature for 1.5 h, the protein bands were visualized using enhanced chemiluminescence (Tanon, China).

### 2.13 Immunohistochemistry

Tissues were fixed with 4% paraformaldehyde, dehydrated, embedded in paraffin and sectioned at 4μm. The sections were deparaffinized, then boiled with citrate solution, and incubated with 3% H_2_O_2_ for 10 min. After antigen retrieval and blocking, the slides were incubated with the indicated antibodies at 4°C overnight. Subsequently, the slides were incubated with the corresponding secondary antibody for 30 min at room temperature and then incubated with the streptavidin peroxidase complex. Staining was performed using a 3,3-diaminobenzidine (DAB) substrate kit for peroxidase reaction and counterstained with hematoxylin. Sections were washed, dehydrated, and sealed with neutral balsam. Finally, an Olympus microscope was used to acquire images.

### 2.13 Statistical analysis

All statistical analyses were conducted using Perl version 5.38.2 and R software version 4.3.1. A P-value of less than 0.05 was used to determine statistical significance. In MR analysis, we employed five methods, including “MR Egger,” “Weighted Median,” “Inverse Variance Weighting (IVW),” “simple mode,” and “weighted mode.” Among these, IVW is the primary method for causal inference due to its superior precision and stability. A P-value of less than 0.05 was considered indicative of a significant correlation between exposure and outcome. We conducted sensitivity and heterogeneity tests based on the Q statistic. The MR-egger regression test was utilized to assess the presence of horizontal pleiotropy based on its intercept term. MR-PRESSO compares the observed distance of all variants to the regression line with the expected distance under the null hypothesis of no horizontal pleiotropy. To evaluate whether the removal of individual influential SNPs influenced the overall estimate of causal effects, leave-one-out analyses were performed.

## 3 Results

### 3.1 Identification of DEGs in PCa

**Figure 1:**
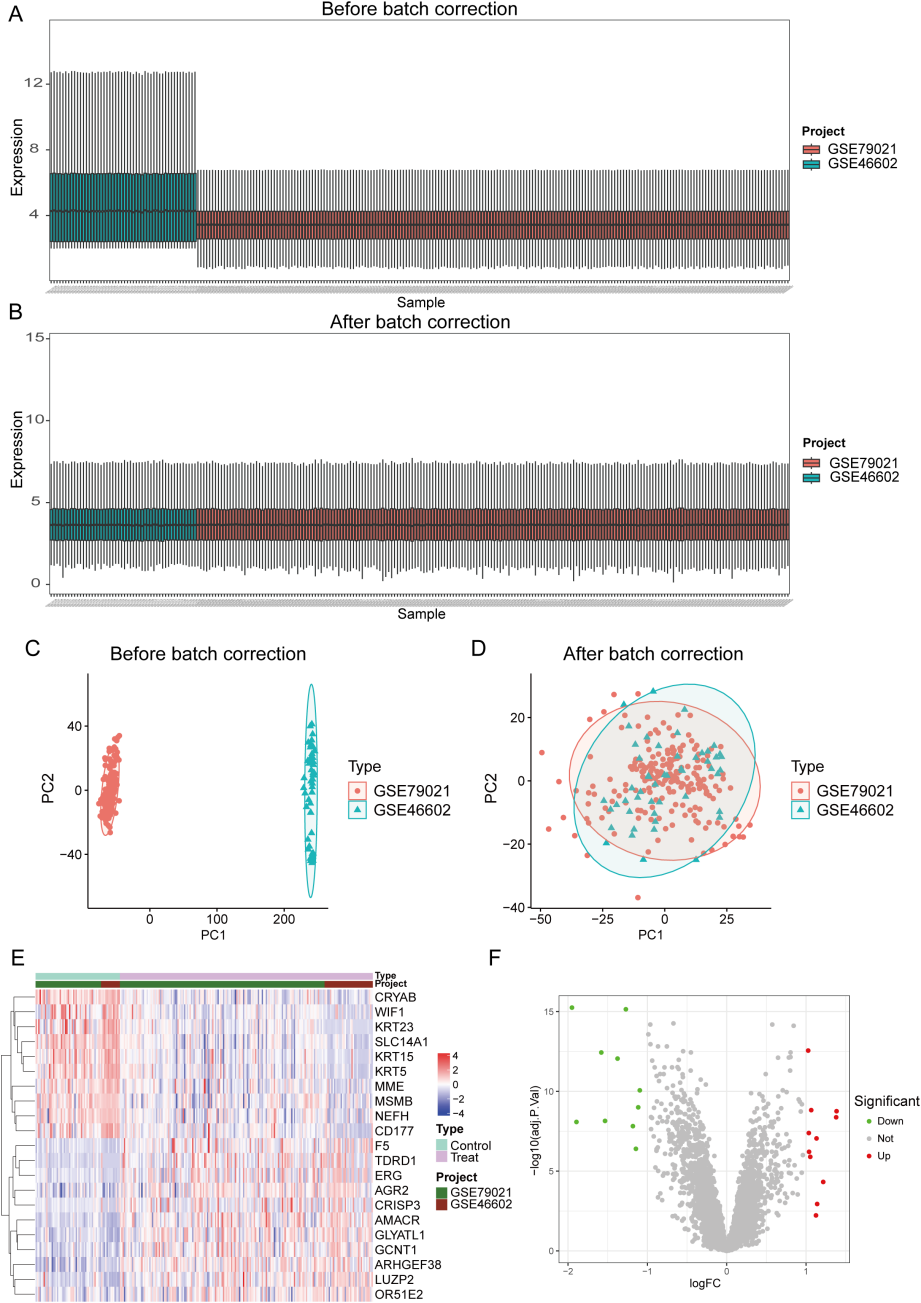
Identification of differential genes in PCa. **(A, C)** The dataset before batch effect removal. **(B, D)** The integrated dataset after batch effect removal. **(E, F)** Heatmaps and volcano plots display the expression of differentially expressed genes in PCa samples and control samples.

DEGs in the GEO database (GSE46602 and GSE79021) were identified in 189 cases of PCa and 63 normal controls using the R package “limma”. Due to batch effects present between different datasets (as shown in **Figures 1A, C**), after removal, the distribution of data across different datasets became significantly more uniform, with medians aligned and both mean and variance showing stronger consistency(as shown in **Figures 1B, D**). This indicates that after adjustment, the integrated data can be considered as a unified batch for subsequent analysis. Based on this, we identified a total of 21 DEGs in the training group, 11 of which were upregulated and 10 were downregulated. The expression levels of the 21 DEGs are displayed in heatmaps and volcano plots (**Figures 1E, F**).

### 3.2 Weighted gene co-expression network analysis and identification of key modules

**Figure 2:**
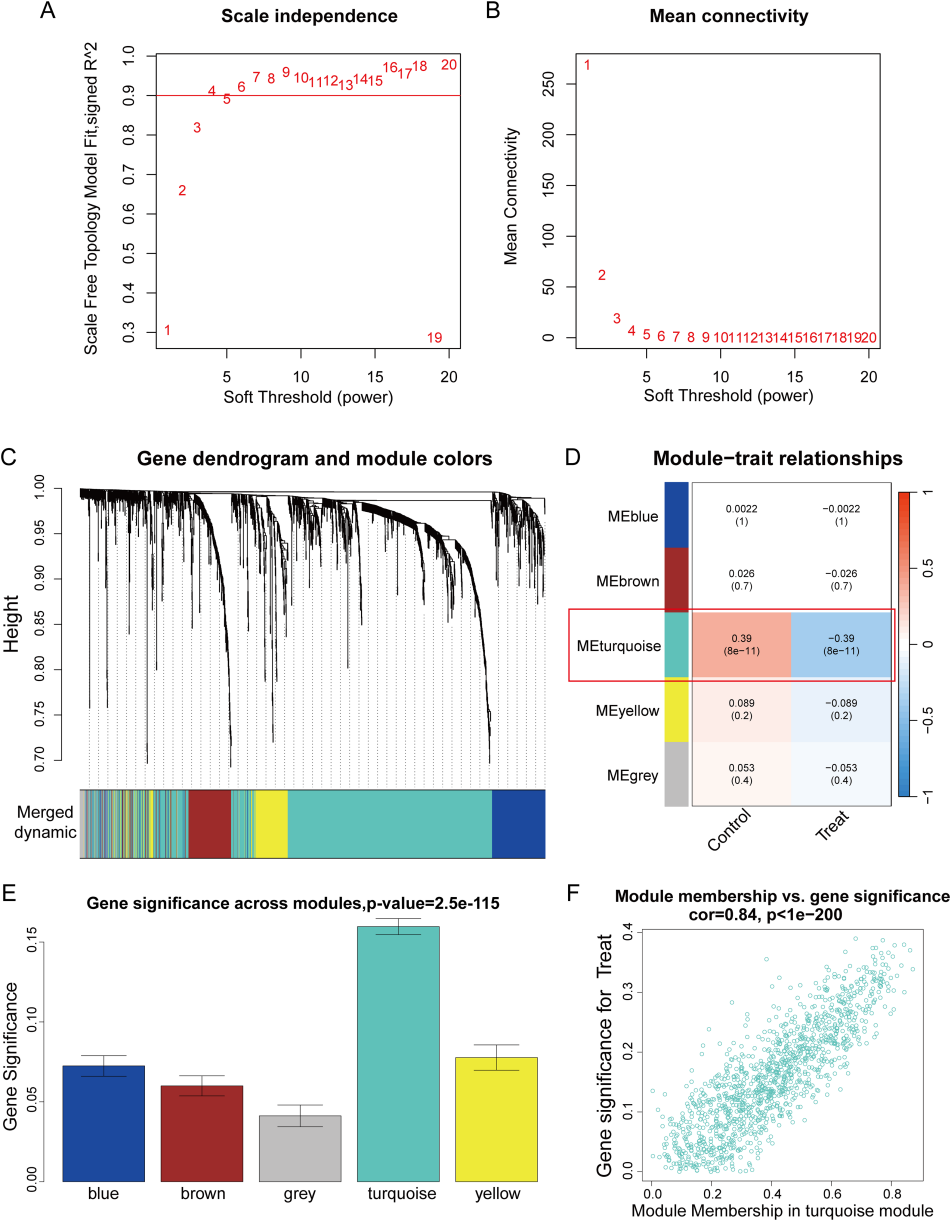
WGCNA analysis. **(A)** The scale-free topology fit index (R²) for soft threshold powers (β) ranging from 1 to 20. **(B)** The mean connectivity for β from 1 to 20. **(C)** Dendrogram of all genes clustered based on topological overlap. **(D)** Correlation heatmap of modules and clinical traits. **(E)** Boxplot of Gene Significance (GS) distribution across five modules. **(F)** Scatterplot of Module Membership (MM) in the turquoise module versus GS for PCa.

We constructed a scale-free co-expression network using WGCNA to identify the most highly correlated modules in PCa. The optimal “soft” threshold β = 4 (scale-free R² = 0.9) was selected based on scale independence and mean connectivity (**Figures 2A, B**). A total of 5 modules with different colors were obtained through dynamic tree shearing (**Figure 2C**). Then, the modules were correlated with clinical characteristics to further identify the driver genes of the most strongly positively correlated module. We ultimately identified the ME turquoise module as being most highly correlated with DCM in PCa (R = 0.39, p < 0.001) (**Figure 2D**). A significant distribution of Gene Significance (GS) was observed across the five modules (**Figure 2E**). Therefore, we selected the turquoise module, which contains 1,195 genes, for further analysis. Additionally, we performed a correlation analysis between module membership (MM) and gene significance (GS), and found a significant positive correlation between them (R = 0.84, p < 0.001; **Figure 2F**).

### 3.3 Functional enrichment analysis

**Figure 3:**
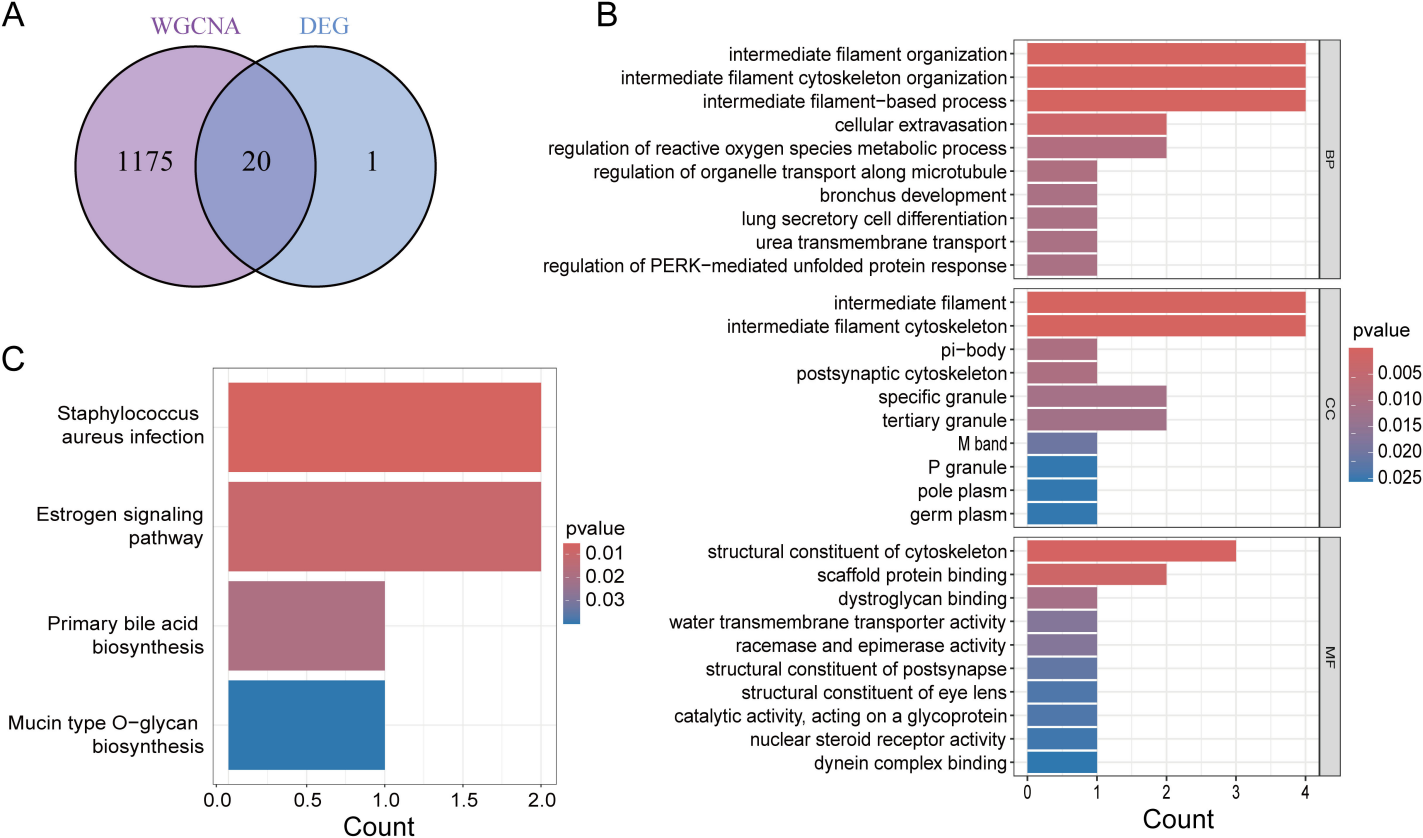
Functional enrichment analysis. **(A)** Venn diagram showing common genes of key module genes and differential genes. **(B)** GO enrichment analysis of Intersecting genes. **(C)** KEGG enrichment analysis of Intersecting genes.

We intersected the DEGs with the WGCNA module genes to obtain a total of 20 genes (**Figure 3A**). To further assess the potential regulatory pathways of these genes in PCa, we conducted GO and KEGG enrichment analyses. The GO analysis indicated that these genes are primarily enriched in the biological process (BP) terms “intermediate filament organization,” “intermediate filament cytoskeleton organization,” and “intermediate filament-based process.” Regarding the cellular component (CC) ontology, these genes are involved in “intermediate filament” and “intermediate filament cytoskeleton.” The molecular function (MF) analysis showed that the genes are enriched in “structural constituent of cytoskeleton” and “scaffold protein binding” (**Figure 3B**). The KEGG enrichment analysis demonstrated that these genes participate in the “Staphylococcus aureus infection” and “Estrogen signaling pathway” (**Figure 3C**).

### 3.4 Identification of candidate core genes through machine learning methods

**Figure 4:**
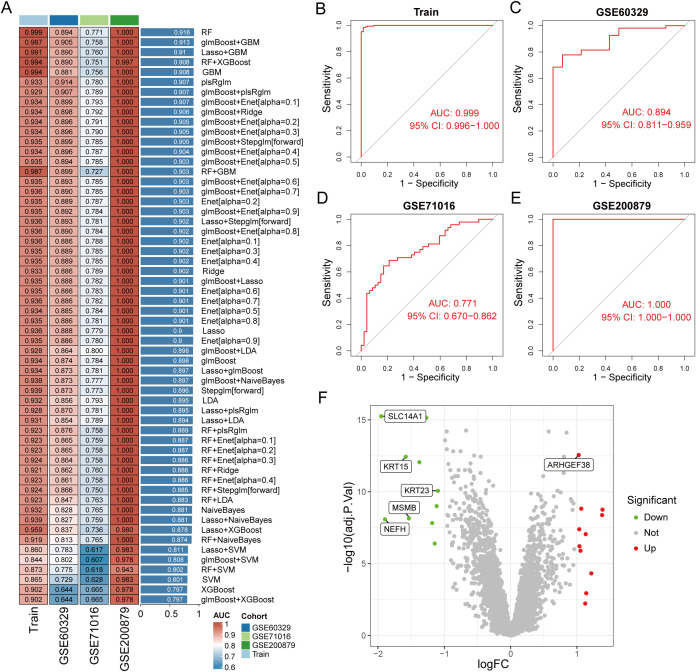
Identification of core genes in prostate cancer using machine learning methods. **(A)** Constructing a prostate cancer diagnostic prediction model with combinations of 113 machine learning algorithms in training and test group. **(B–E)** ROC curves of RF model for training and test group. **(F)** Volcano plot of the 6 core genes identified by the RF model.

To construct a diagnostic model for PCa-related genes, we utilized a combination of 113 machine learning algorithms to analyze the previously selected 20 genes. In the training group dataset, we calculated that “RF” produced the best predictive performance among the 113 tested algorithms, and this model also performed well in the test group dataset. Furthermore, we calculated the AUC values for each model in both the training and validation groups, with the RF machine learning algorithm achieving the highest AUC value (**Figure 4A**). Therefore, we identified RF as the optimal diagnostic model. Additionally, we plotted the ROC curves for the RF model in the training and validation groups across three datasets (**Figures 4B–E**). The RF model identified six genes (SLC14A1, ARHGEF38, NEFH, MSMB, KRT23, KRT15) as the final validated core genes (**Figure 4F**).

### 3.5 Diagnostic assessment and validation of core genes

**Figure 5:**
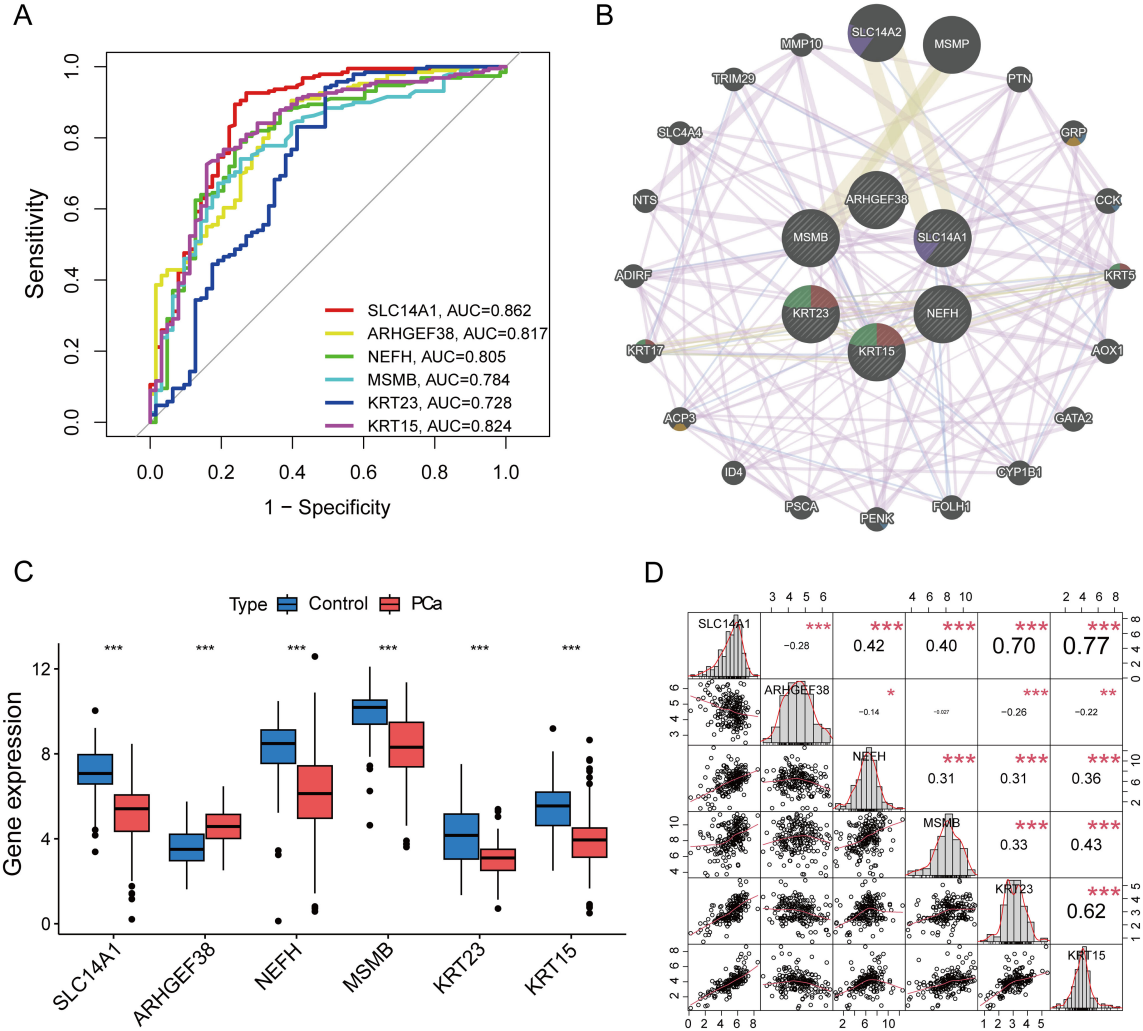
Assessment of Diagnostic Value of Core Genes and Correlation Analysis. **(A)** The ROC curves for the diagnostic validity of 6 core genes. **(B)** PPI network among 6 core genes.**(C)** The expression of 6 core genes in PCa and control tissues. **(D)** Correlation analysis among 6 genes. *P < 0.05, **P < 0.01, ***P < 0.001 compared to control.

To assess the diagnostic value of the six candidate core genes selected, we established ROC curves to evaluate the diagnostic specificity and sensitivity of each gene. The results are as follows: SLC14A1 (AUC 0.862), ARHGEF38 (AUC 0.817), NEFH (AUC 0.805), MSMB (AUC 0.784), KRT23 (AUC 0.728), KRT15 (AUC 0.824), all candidate genes have a high diagnostic value for PCa (**Figure 5A**). To delve deeper into the biological significance of these six core genes, we utilized GeneMANIA to analyze the relationships and interaction patterns between these genes and other closely related genes. we found 20 genes that are closely related to these 6 genes (**Figure 5B**). Next, we applied the Gene Set Variation Analysis (GSVA) method to analyze the signaling pathways in the KEGG database to assess the enrichment of each gene in these pathways. We found that the genes SLC14A1, MSMB, KRT23, and KRT15 are primarily enriched in “prostate cancer” signaling pathway (**Supplementary Figure S1**). We also analyzed the expression level differences of these core genes in PCa patients and normal populations. The results showed that SLC14A1, NEFH, MSMB, KRT23, and KRT15 are underexpressed in PCa, while ARHGEF38 is highly expressed in PCa (**Figure 5C**). In addition, we also analyzed the correlations between these core genes and found that SLC14A1, NEFH, MSMB, KRT23, and KRT15 genes are all positively correlated with each other, while ARHGEF38 is negatively correlated with the other five genes (**Figure 5D**).

### 3.6 Immune cell infiltration analysis

**Figure 6:**
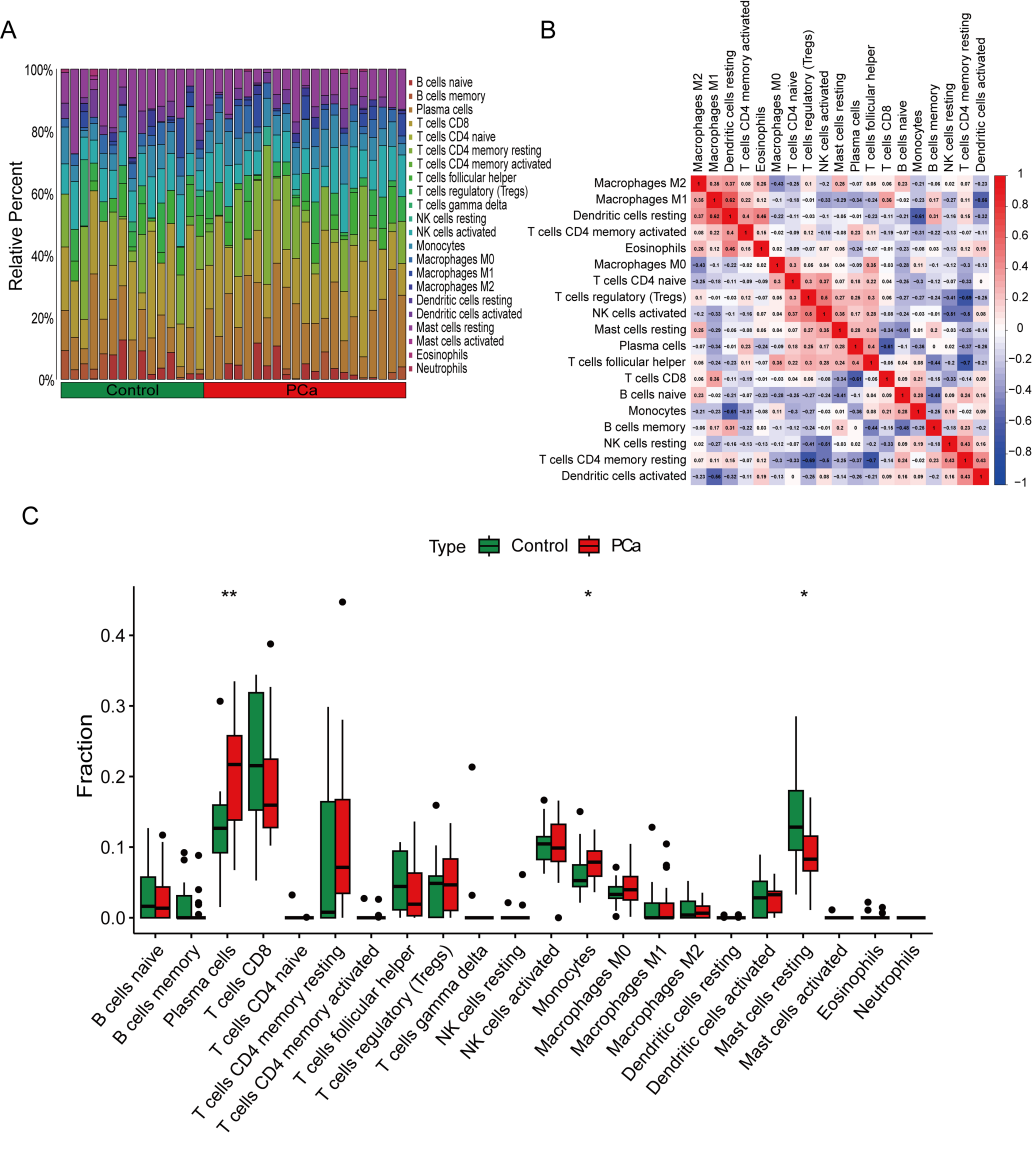
Immune cell infiltration analysis between PCa and control. **(A)** The box-plot diagram indicating the proportion of 22 kinds of immune cells between PCa and normal controls. **(B)** The heat map illustrates the correlation among 22 different immune cell populations.**(C)** The box plot shows the differences in immune infiltration between prostate cancer and normal controls. *P < 0.05, **P < 0.01 compared to control.

We used the CIBERSORT algorithm to evaluate the composition and abundance of 22 types of immune cell infiltration in the training group (**Figure 6A**). In addition, the correlation analysis of the 22 types of immune cells showed that Dendritic cells resting were positively correlated with Macrophages M1 (r = 0.62), T cells regulatory were positively correlated with NK cells activated (r = 0.50), while T cells CD4 memory resting were negatively correlated with T cells follicular helper (r = −0.70) (**Figure 6B**). The box plots showed that compared to the control group, PCa patients had higher levels of Plasma cells and Monocytes, while the levels of Mast cells resting were lower (**Figure 6C**).

### 3.7 Correlation analysis between PCa-associated core genes and immune infiltrating cells

**Figure 7:**
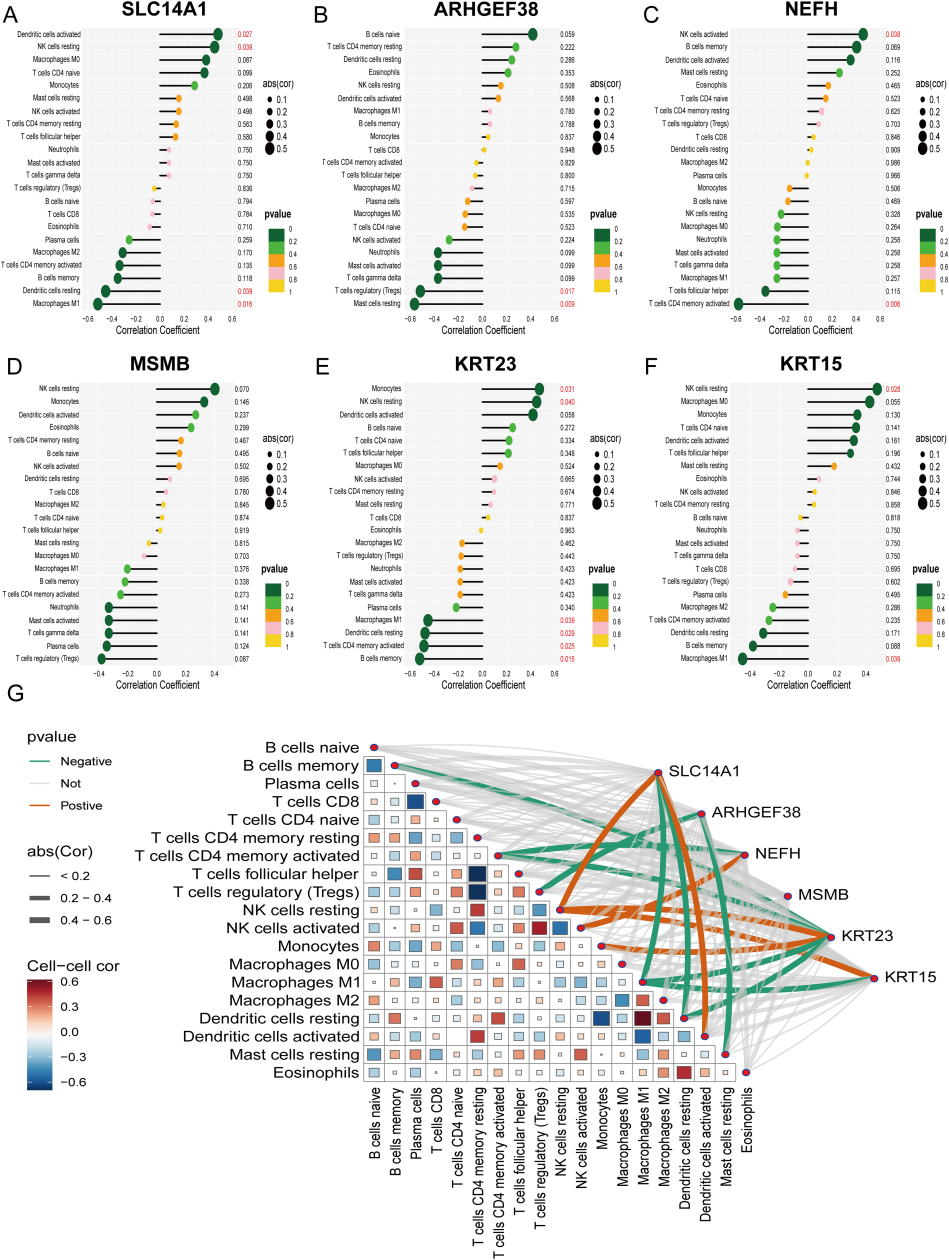
Correlation analysis of 6 PCa-related core genes with infiltrating immune cells. **(A–F)** Correlation between SLC14A1, ARHGEF38, NEFH, MSMB, KRT23, KRT15, and infiltrating immune cells. **(G)** A visualization chart of the correlations between 6 core genes and the infiltration of 22 types of immune cells.

To validate the relationship between the selected core genes and immune cell infiltration, we conducted a correlation analysis. The results indicated that SLC14A1 is positively correlated with Dendritic cells activated (R = 0.48, P = 0.027) and NK cells resting (R = 0.45, P = 0.039), and negatively correlated with Dendritic cells resting (R = -0.45, P = 0.039) and Macrophages M1 (R = -0.52, P = 0.016) (**Figure 7A**); ARHGEF38 is negatively correlated with T cells regulatory (Tregs) (R = -0.52, P = 0.017) and Mast cells resting (R = -0.56, P = 0.009) (**Figure 7B**); NEFH is positively correlated with NK cells activated (R = 0.46, P = 0.038) and negatively correlated with T cells CD4 memory activated (R = -0.58, P = 0.006) (**Figure 7C**); MSMB shows no significant correlation with immune cell infiltration (**Figure 7D**); KRT23 is positively correlated with Monocytes (R = 0.47, P = 0.031) and NK cells resting (R = 0.45, P = 0.040), and negatively correlated with Macrophages M1 (R = -0.45, P = 0.039), Dendritic cells resting (R = -0.48, P = 0.029), T cells CD4 memory activated (R = -0.49, P = 0.025), and B cells memory (R = -0.52, P = 0.015) (**Figure 7E**); KRT15 is positively correlated with NK cells resting (R = 0.48, P = 0.028) and negatively correlated with Macrophages M1 (R = -0.45, P = 0.039) (**Figure 7F**). Additionally, we visualized the correlations between the expression of all core genes and the infiltration of 22 immune cells (**Figure 7G**). These findings suggest that the associated core genes may contribute to PCa by influencing immune cell infiltration.

### 3.8 MR analysis of the association between core genes and PCa

**Figure 8:**
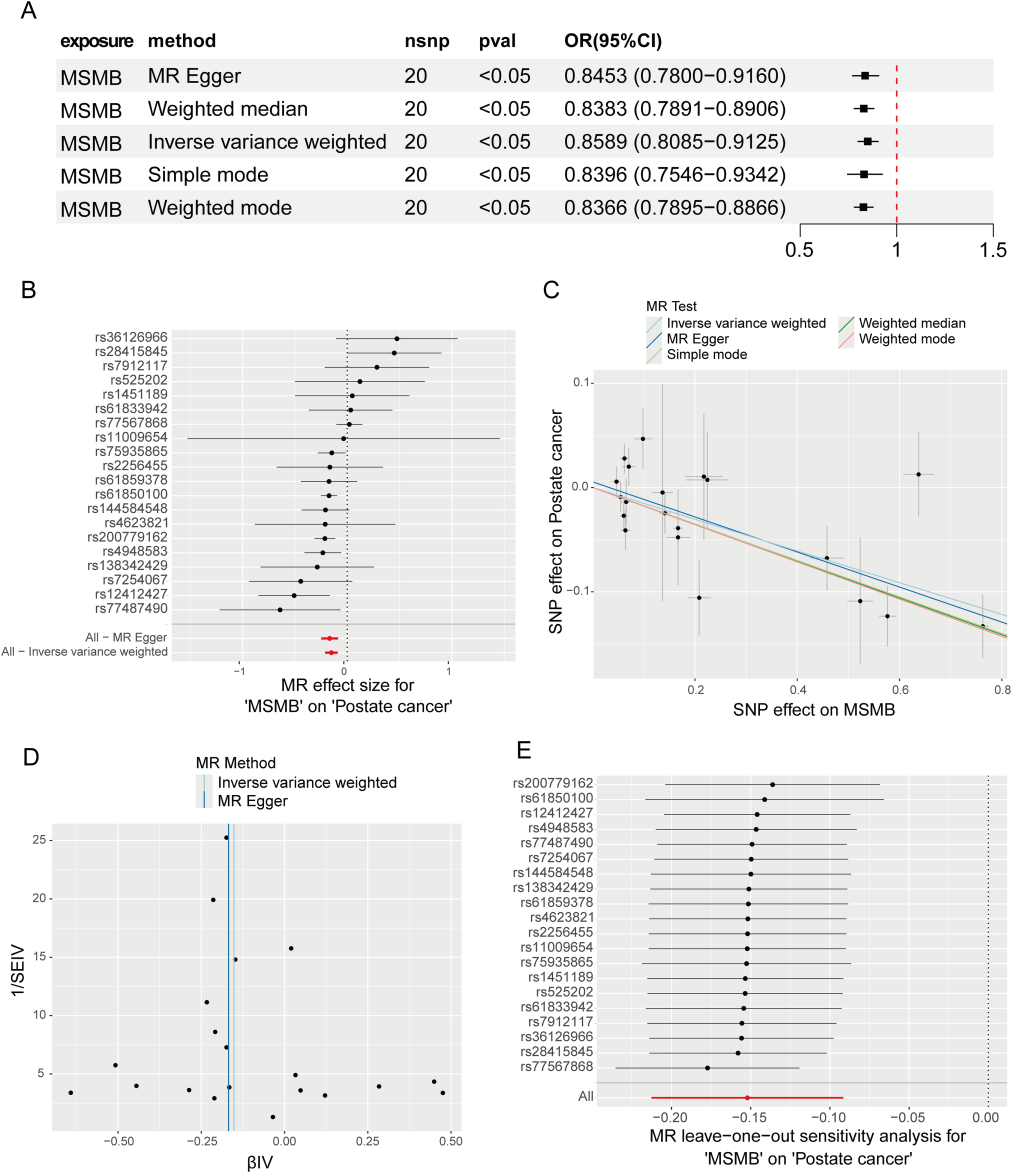
Mendelian randomization study results on the relationship between MSMB and PCa. **(A)** MR analysis demonstrated the causal relationship of MSMB pQTL on PCa. **(B–E)** Forest plot, Funnel plot, Scatter plot and Leave-one-out analysis of the causal association between MSMB pQTL and PCa.

In our study, we initially conducted a two-sample MR analysis using eQTL data of six core genes combined with PCa data. Unfortunately, this analysis did not reveal any causal associations between these genes and PCa. Subsequently, we turned to another round of two-sample MR analysis using the pQTL data of these core genes along with PCa data. In this analysis, we found that only the MSMB pQTL had a significant causal relationship with the incidence of PCa (OR: 0.8589, 95% CI: 0.8085-0.9125, P < 0.05) (**Figure 8A**). This indicates that the expression of MSMB is a major protective factor in the pathogenesis of PCa, while the other core genes may only appear as biomarkers after the occurrence of PCa. The forest plot shows the effect sizes of various SNPs in the MSMB pQTL (**Figure 8B**). The scatter plot shows the regression trends of the five methods, all of which are consistent (**Figure 8C**). The funnel plot shows the distribution of causal effects (**Figure 8D**). The leave-one-out analysis reveals the impact of individual SNPs on the overall causal estimate (**Figure 8E**). The MR-Egger intercept analysis for the MSMB pQTL did not detect any potential horizontal pleiotropy (P = 0.55), indicating that the instrumental variables do not significantly affect the outcome through pathways other than the exposure. In the Cochran Q test for heterogeneity, heterogeneity was observed in the MSMB pQTL data (P = 0.013). This may be due to IVs originating from different analysis platforms, different studies or datasets, and different populations.

### 3.9 Validation of core gene expression in prostate cancer and adjacent non-cancerous tissues

**Figure 9:**
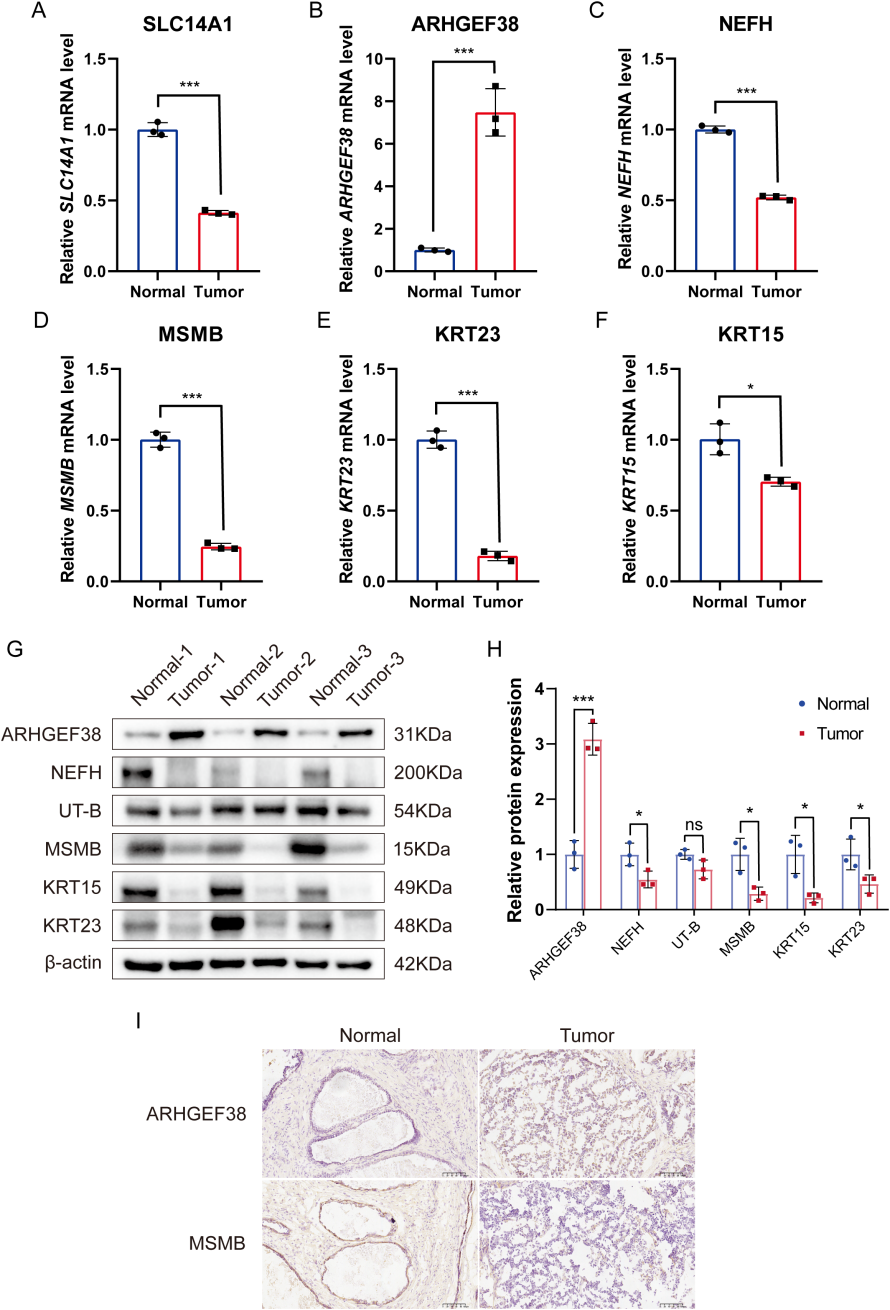
Validation of mRNA and protein expression levels of six core genes in PCa tumor tissues and adjacent non-cancerous tissues. **(A–F)** mRNA expression levels of SLC14A1, ARHGEF38, NEFH, MSMB, KRT23, and KRT15 in PCa tumor tissues and adjacent non-cancerous tissues, as determined by qRT-PCR. (n=3). **(G)** Protein expression levels of UT-B (encoded by SLC14A1), ARHGEF38, NEFH, MSMB, KRT23, and KRT15 in PCa tumor tissues and adjacent non-cancerous tissues, as determined by Western blot. **(H)** Quantitative analysis of Western blot results. **(I)** Immunohistochemistry (IHC) analysis of ARHGEF38 and MSMB protein expression in PCa tumor tissues and adjacent non-cancerous tissues. Data are presented as mean ± SEM. ns P>0.05, *P < 0.05, ***P < 0.001 compared to normal tissues.

We measured the mRNA expression levels of six core genes in PCa tumor tissues and their corresponding para-cancerous tissues. Our results revealed that, compared to para-cancerous tissues, the expression levels of SLC14A1, NEFH, MSMB, KRT23, and KRT15 were significantly downregulated in PCa tumor tissues, while ARHGEF38 expression was notably upregulated, consistent with our initial predictions (**Figures 9A–F**). To further validate these findings at the protein level, we conducted Western blot analysis on tumor tissues and adjacent normal tissues from PCa patients. The results demonstrated that ARHGEF38 protein expression was significantly elevated in tumor tissues. In contrast, the expression levels of UT-B (encoded by SLC14A1), NEFH, MSMB, KRT23, and KRT15 were reduced in tumor tissues compared to adjacent normal tissues, although the decrease in UT-B did not reach statistical significance (**Figure 9G**). Quantitative analysis of the Western blot results is presented in **Figure 9H**, further supporting the observed protein expression trends.

Additionally, immunohistochemistry (IHC) was performed to evaluate the localization and expression levels of two key proteins in PCa and para-cancerous tissues. The IHC results revealed that ARHGEF38 protein was highly expressed in tumor tissues, while MSMB exhibited reduced expression in tumor tissues compared to para-cancerous tissues (**Figure 9I**). These findings were consistent with the mRNA and protein expression patterns observed in qRT-PCR and Western blot analyses.

## 4 Discussion

PCa is one of the most common malignant tumors in the male reproductive system and is the fifth leading cause of cancer-related deaths in men. Its pathogenesis is complex, involving a variety of factors such as genetics, environment, and lifestyle. Although the causes of PCa are not yet clear, early prevention and treatment remain key to improving prognosis and prolonging survival. PCa-related biomarkers play an important role in the early diagnosis, prognosis assessment, and monitoring of treatment response in PCa (19). In recent years, with the advancement of molecular biology and genetic technology, more and more PCa-related biomarkers have been discovered and applied in clinical and research settings. For example, Srikantan et al. (20)identified a prostate-specific gene PCGEM1 that is expressed only in human prostate tissue and is upregulated in the vast majority of tumor tissues by comparative analysis of differentially expressed genes between normal prostate tissue and PCa tissue. This indicates that PCGEM1 may be involved in the occurrence and progression of PCa. Wei et al. (21) assessed the value of urine prostate cancer antigen 3 (PCA3) for screening PCa in men, and the results showed that urine PCA3 plays an important role in reducing the medical burden of men undergoing repeat prostate biopsies, and for men who have not undergone a biopsy, a high PCA3 score significantly increases the likelihood of detecting cancer in the initial prostate biopsy. We obtained five datasets from the GEO database, of which two were combined into the training group, and the remaining three were combined into the test group. In the training group, we identified 21 DEGs, of which 11 genes were upregulated and 10 genes were downregulated. Additionally, KEGG enrichment analysis revealed that these differentially expressed genes are involved in pathways related to “Staphylococcus aureus infection.” The identification of shared genetic pathways between PCa and Staphylococcus aureus infection suggests potential interactions between microbial infections and PCa progression. For example, S. aureus may play a role in modulating the tumor immune microenvironment, thereby influencing prostate cancer pathogenesis (22, 23). For example, S. aureus may influence the progression and metastasis of prostate cancer by modulating the tumor immune microenvironment. These genetic overlaps may play an important role in the regulation of microbial infections and the immune microenvironment. Further studies are needed to explore these mechanisms in detail.

Subsequently, we used WGCNA and 12 machine learning methods to screen for PCa-related core genes. Ultimately, we identified Random Forest (RF) as the optimal machine learning method and discerned six core genes from it, including SLC14A1, ARHGEF38, NEFH, MSMB, KRT23, and KRT15. To validate whether these genes could act as diagnostic biomarkers, we analyzed their diagnostic value in PCa. The results demonstrated that all of these genes exhibited certain diagnostic potential, with AUC values all exceeding 0.7.

Previous studies have indicated that immune cell infiltration is closely related to the development and prognosis of PCa. In the tumor microenvironment of PCa, the mechanism and interaction of immune cells are very complex, and they play significant roles in the occurrence and progression of cancer (24). Our study used CIBERSORT to evaluate the infiltration of immune cells in PCa, and the results showed an increase in the expression of Plasma cells and Monocytes, while the expression of Mast cells resting decreased in PCa patients. In addition, we analyzed the relationship between the six core genes and PCa-related immune cells, and the results revealed that SLC14A1, NEFH, KRT15, and KRT23 were significantly correlated with NK cells. Furthermore, SLC14A1, KRT15, and KRT23 were significantly associated with Macrophages M1. Natural Killer (NK) cells are a subset of lymphocytes that play a central role in the innate immune response to tumors and infections. NK cells can detect changes in the expression of self MHC-I molecules on the surface of autologous cells and maintain cytotoxic activity against tumors with high expression of MHC-I (25). A follow-up study over 11 years indicated that low NK cell cytotoxic activity is associated with an increased risk of cancer (26). Pasero et al. (27) retrospectively analyzed natural killer (NK) cells in the peripheral blood of 39 patients with metastatic PCa and found that patients with longer survival and castration-resistant time showed high expression of NK cell receptors (NKp46 and NKp30). Seki et al. (28) demonstrated that NK cells can target PCa stem-like cells through a pathway, thereby inhibiting the occurrence, progression, metastasis, and recurrence of PCa. Tumor-associated macrophages are one of the major types of immune cells in the TME, and an increase in their density is associated with poor prognosis in PCa. Kainulainen et al. (29) showed that the secreted factors of M1 macrophages can upregulate the plasticity of PCa stem cells through the NF-κB signaling pathway, thereby affecting the progression and drug resistance of PCa. Hadimani et al. (30) analyzed the expression of CD68 and CD163 in tumor tissues from 62 patients with PCa to evaluate the expression of tumor-associated macrophages M1 and M2 in PCa and explore their association with tumor stage. The results showed that CD68 expression was associated with a good prognosis with fewer lymph node and distant metastases, while CD163 expression was associated with a poor prognosis and an increased likelihood of lymph node and distant metastases. These studies, including our own, indicate that various types of immune cells play important roles in PCa, providing new insights for immunotherapy of PCa.

Although we have identified 6 core genes associated with PCa, the genetic associations of these genes with PCa still need to be studied in depth. Therefore, I employed Mendelian randomization analysis to further explore the potential causal effects of these genetic variants on the risk of developing PCa. Our study did not find any significant association between the eQTL data of any genes and PCa; however, we did find that the pQTL data of the MSMB gene showed a significantly negative correlation with the occurrence of PCa. This finding suggests that the expression level of the MSMB gene may have a significant impact on the risk of developing PCa.

In this study, we systematically validated the expression patterns of six core genes (SLC14A1, ARHGEF38, NEFH, MSMB, KRT23, and KRT15) in PCa tissues and adjacent non-cancerous tissues using qRT-PCR, Western blot, and IHC. Our qRT-PCR results demonstrated that SLC14A1, NEFH, MSMB, KRT23, and KRT15 were significantly downregulated in PCa tissues, while ARHGEF38 was markedly upregulated. These findings were further corroborated at the protein level through Western blot analysis, which revealed consistent expression trends for ARHGEF38, NEFH, MSMB, KRT23, and KRT15. Although the reduction in UT-B (encoded by SLC14A1) protein expression did not reach statistical significance, the overall trend aligned with the mRNA data, suggesting a potential role of these genes in PCa pathogenesis. This lack of statistical significance for UT-B may be attributed to the relatively small sample size, which could limit the power to detect subtle differences in protein expression. Future studies with larger cohorts are needed to confirm these findings and further explore the functional implications of UT-B in PCa.

To strengthen our findings, we performed IHC analysis for two key proteins, ARHGEF38 and MSMB. The IHC results confirmed that ARHGEF38 protein was highly expressed in tumor tissues, while MSMB expression was markedly reduced, consistent with the qRT-PCR and Western blot data. These results not only validate the dysregulation of these genes at both mRNA and protein levels but also highlight their potential as biomarkers or therapeutic targets in PCa. The consistent downregulation of MSMB, a known tumor suppressor in prostate cancer, further supports its role in inhibiting tumor progression. Conversely, the upregulation of ARHGEF38, a gene implicated in cell signaling and cytoskeletal reorganization, suggests its potential involvement in promoting tumor growth and metastasis.

This study indicates that the genes SLC14A1, ARHGEF38, NEFH, MSMB, KRT23, and KRT15 hold significant diagnostic value in the onset and progression of PCa. SLC14A1, a member of the urea transporter family and a type B urea transporter (UT-B), plays a crucial role in regulating urine concentration (31). Furthermore, the SLC14A1 gene is also considered a novel tumor suppressor for urothelial carcinoma, and in PCa, the downregulation of its expression promotes tumor progression by activating the CDK1/CCNB1 and mTOR pathways and is closely associated with biochemical recurrence (BCR) of PCa (32–34). ARHGEF38 is a component of the human Rho guanine nucleotide exchange factor family, which plays an important role in cell migration and tumorigenesis. Studies have shown that knocking down the expression of ARHGEF38 can inhibit the proliferation, migration, and invasion of PCa cells, suggesting that ARHGEF38 may promote the progression of PCa (35). The protein encoded by the NEFH gene is a key component of the neurofilament cytoskeleton and belongs to the family of intermediate filament proteins; its gene mutations are associated with various neurological diseases (36). MSMB (β-microseminoprotein), primarily expressed in the prostate, is an abundant component in semen and is considered one of the biomarkers for PCa, holding significant value in the diagnosis and treatment of PCa (37). KRT23 is a member of the keratin family and an acidic keratin expressed in various cancer types, including pancreatic, colorectal, and hepatocellular carcinomas (38–40). Research by Wang et al. (41) indicates that the knockout of the KRT23 gene can enhance the metastatic and invasive capabilities of PCa DU145 cells. KRT15, also a member of the keratin family, is an intermediate filament protein expressed in various epithelial cells and plays an essential role in maintaining cellular structural stability, proliferation, and differentiation. Research by Xiao et al. (42) shows that KRT15 is expressed at lower levels in PCa tissues and cell lines (LNCaP, DU145, and PC3), and the low expression of KRT15 is correlated with higher pathological staging and Gleason scores. Our study results are consistent with existing research, indicating that these genes may significantly impact the development of PCa and can be used for early diagnosis. In particular, MSMB shows a strong causal relationship in the Mendelian randomization analysis related to the occurrence of PCa and may become an important protective factor for PCa. However, it is important to clarify that these biomarkers are not intended to replace biopsy as the gold standard for definitive diagnosis. Instead, they aim to serve as non-invasive supplementary tools to reduce the reliance on biopsy, particularly in cases where PSA levels are ambiguous or patients are at high risk of complications from invasive procedures. By integrating these biomarkers into existing diagnostic workflows, clinicians can make more informed decisions about whether a biopsy is necessary, thereby minimizing unnecessary invasive interventions.

This study has certain limitations. Firstly, the sample size of the datasets used in this study is limited, which means that our findings need to be validated in larger datasets and clinical trials to enhance the reliability of the research results. Secondly, while our qRT-PCR, Western blot and IHC experiments confirmed the differential expression of the identified biomarkers, further functional studies are needed to elucidate their roles in prostate cancer progression and immune modulation. Future research should include *in vitro* experiments, such as gene knockdown or overexpression, and *in vivo* studies using animal models to validate the mechanistic roles of these biomarkers. Thirdly, our MR analysis suggests that MSMB is a protective factor for PCa; however, the presence of heterogeneity may limit the strength and reliability of the research conclusions. This could be due to the IVs originating from different analysis platforms, different studies or datasets, and different populations. Future studies should employ more stringent criteria for selecting instrumental variables to enhance the robustness of MR findings.

In summary, our study has made a significant contribution to prostate cancer research by combining advanced computational methods with experimental validation, especially in the context of personalized treatment strategies. The genes SLC14A1, ARHGEF38, NEFH, MSMB, KRT23, and KRT15 have been identified as biomarkers associated with PCa. Mendelian randomization analysis indicates that MSMB is an important protective factor related to the occurrence of PCa. These biomarkers could be used to stratify patients based on their risk profiles, enabling more targeted and effective interventions. Additionally, these biomarkers may serve as non-invasive tools for early diagnosis, reducing the need for invasive procedures like biopsies. However, addressing the noted limitations, including expanding functional validation and exploring comparative analyses with PSA, will be essential for translating these findings into clinical practice. Future studies should include longitudinal analyses to assess changes in biomarker expression across different stages of prostate cancer. Such analyses could validate the prognostic utility of these biomarkers and provide insights into their roles in disease progression.

## Data Availability

The original contributions presented in the study are included in the article/**Supplementary Material**. Further inquiries can be directed to the corresponding authors.
