# Supplementary figures and images for: WGCNA-ML-MR integration: uncovering immune-related genes in prostate cancer
Source: Front Oncol. 2025 Apr 7;15:1534612. doi: 10.3389/fonc.2025.1534612 (PMC12009700; doi:10.3389/fonc.2025.1534612)

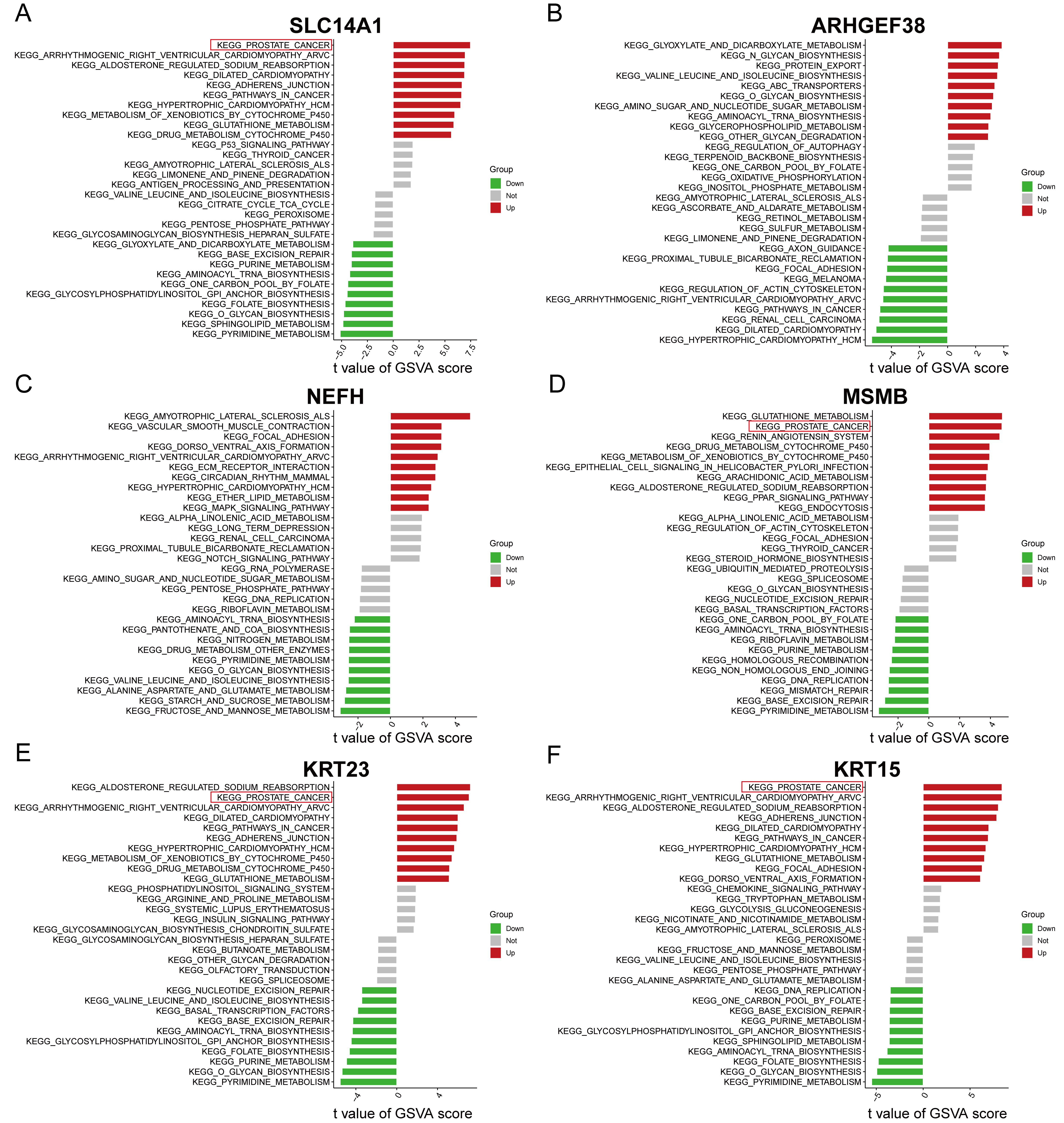

Supplement: Supplementary file 1 [file Image1.tif]
