# Supplementary material for: WGCNA-ML-MR integration: uncovering immune-related genes in prostate cancer
Source: Front Oncol. 2025 Apr 7;15:1534612. doi: 10.3389/fonc.2025.1534612 (PMC12009700; doi:10.3389/fonc.2025.1534612)
Supplement: Supplementary file 2 [file Table1.docx]

**WGCNA-ML-MR Integration: Uncovering Immune-Related Genes in Prostate Cancer**

**Supplementary Table 1. The primers used in the present study.**

| Gene | Primer | Sequence (5'-3') |
| --- | --- | --- |
| SLC14A1 | Forward | AATGTTCATGGCGCTCAC |
|  | Reverse | ATGCCGACTCCAAGATAG |
| ARHGEF38 | Forward | TGTTAGGGAAGTGGTTCA |
|  | Reverse | CACGGACTCAATGTTGCT |
| NEFH | Forward | GCCGACATTGCCTCCTAC |
|  | Reverse | GAGCCATCTTGACATTGAGC |
| MSMB | Forward | TTTCTACACCTGTGGGTTAT |
|  | Reverse | CTTCTTCTCCACCACGAT |
| KRT23 | Forward | GGATGGCAGTGGATGACT |
|  | Reverse | CACCTCCTGTTCTAGGTCTGTT |
| KRT15 | Forward | TCCGATGCGAGATGGAGG |
|  | Reverse | GAGCAGGCTGCGGTAAGT |
